# Prevalence of Thyroid Nodules in China: A Health Examination Cohort-Based Study

**DOI:** 10.3389/fendo.2021.676144

**Published:** 2021-05-26

**Authors:** Yunhai Li, Cheng Jin, Jie Li, Mingkun Tong, Mengxue Wang, Jiefeng Huang, Yi Ning, Guosheng Ren

**Affiliations:** ^1^ Department of Endocrine and Breast Surgery, The First Affiliated Hospital of Chongqing Medical University, Chongqing, China; ^2^ Meinian Institute of Health, Beijing, China; ^3^ Peking University Health Science Center, Meinian Public Health Research Institute, Beijing, China; ^4^ Chongqing Key Laboratory of Molecular Oncology and Epigenetics, The First Affiliated Hospital of Chongqing Medical University, Chongqing, China

**Keywords:** thyroid nodes, prevalence, risk factor, ultrasonography, China

## Abstract

**Background:**

Thyroid nodules are a common clinical problem and some are potentially cancerous; however, little is known about the prevalence of thyroid nodules in China. The objective of this study was to investigate the prevalence of thyroid nodules in a healthy Chinese population.

**Methods:**

We reviewed electronic medical records of 13,178,313 participants from 30 provinces and regions who received health examinations and underwent thyroid ultrasound at Meinian Onehealth Healthcare in 2017. Among them, 6,192,357 were excluded based on predefined criteria. All thyroid nodules were diagnosed by ultrasonography, and standardized protocols were adopted for data collection, quality control, and data management.

**Results:**

A total of 6,985,956 participants (mean age: 42.1 ± 13.1 years) were included in this study. The overall prevalence of thyroid nodules was 36.9% (95% CI, 35.7%–38.1%; age- and sex-standardized prevalence 38.0% [95% CI, 37.0%–39.1%]). The prevalence of thyroid nodules in females (44.7% [95% CI, 43.4%–45.9%], age-standardized prevalence: 45.2% [95% CI, 44.1%–46.4%]) was significantly higher than that in males (29.9% [95% CI, 28.8%–31.0%], age-standardized prevalence 31.2% [95% CI, 30.1%–32.2%]; *P* < 0.001). The prevalence of thyroid nodules decreased from <18 to 25 years, while increased with age over 25 years old. The top three provinces with the highest prevalence of thyroid nodules were Jilin (47.6%), Liaoning (44.8%), and Shandong (43.9%), whereas Guizhou (23.9%), Chongqing (26.2%), and Shaanxi (26.4%) had the lowest prevalence. Females had more than 10% higher rates of thyroid nodules than males in all included provinces and regions, except for Tianjin (8.0%). Based on the geographical regions of China, the northeast had the highest prevalence (46.8% [95% CI, 44.1%–49.2%]), whereas northwest had the lowest prevalence (28.9% [95% CI, 26.9%–31.6%]. Based on multivariable logistic regression analysis, factors including age, gender, body mass index, systolic blood pressure, diastolic blood pressure, uric acid, fasting blood glucose, triglycerides, high-density lipoproteins, and low-density lipoproteins were significantly associated with the presence of thyroid nodules.

**Conclusion:**

This study provides the first nationwide analysis of the prevalence of thyroid nodules in China. Our results showed that the prevalence of thyroid nodules was high in health screening Chinese people with regional-specific patterns.

## Introduction

Thyroid nodules are defined as discrete lesions within the thyroid gland that are radiologically distinct from surrounding thyroid tissues (1). The diagnosis of thyroid nodules has become a common event in clinical practice worldwide. Currently, thyroid ultrasound plays a central role in the evaluation and management of thyroid nodules (2). With the increased use of high-resolution ultrasonography, thyroid nodules have been reported in 19%–67% of randomly selected individuals (3). Discovery of a thyroid nodule may be stressful for patients; however, more than 90% of thyroid nodules are benign lesions without symptoms (4). Moreover, a majority of nodules remain stable with regard to size during follow-up (4, 5).

Thyroid nodules are mainly derived from thyroid follicular cells; therefore, benign follicular nodules are the most common lesions. Follicular cell-derived thyroid cancers include papillary (80%–85%), follicular (10%–15%), poorly differentiated (<2%), and undifferentiated (anaplastic; <2%) (6). Thyroid nodule patients with some clinical factors, such as history of childhood irradiation, family history of thyroid cancer in a first-degree relative, male sex, and rapid nodule growth, are associated with increased risk of malignancy (7, 8). Although thyroid cancer screening may lead to earlier diagnosis, there is not adequate evidence to conclude that this would reduce morbidity or mortality (9, 10). Fine-needle aspiration cytology is the most sensitive and cost-effective method of identifying suspicious lesions that require surgery (1, 11). However, there is uncertainty concerning sonographically unsuspicious nodules and benign nodules indicated by fine-needle aspiration regarding the appropriate frequency of follow-up ultrasonography and the repetition of fine-needle aspiration. Overdiagnosis and overtreatment has become a challenge in the current management of thyroid nodules.

The prevalence of thyroid nodules in China has varied from 10.12%–46.56% among different studies (12). However, it is difficult to estimate the real prevalence of thyroid nodules, as China encompasses a big territorial area with diverse environments and a huge population composed of multiple races. In this study, we investigated the prevalence of thyroid nodules in China nationwide based on a health examination cohort with a large sample of 6,985,956 individuals.

## Methods

### Study Cohort

**Figure 1:**
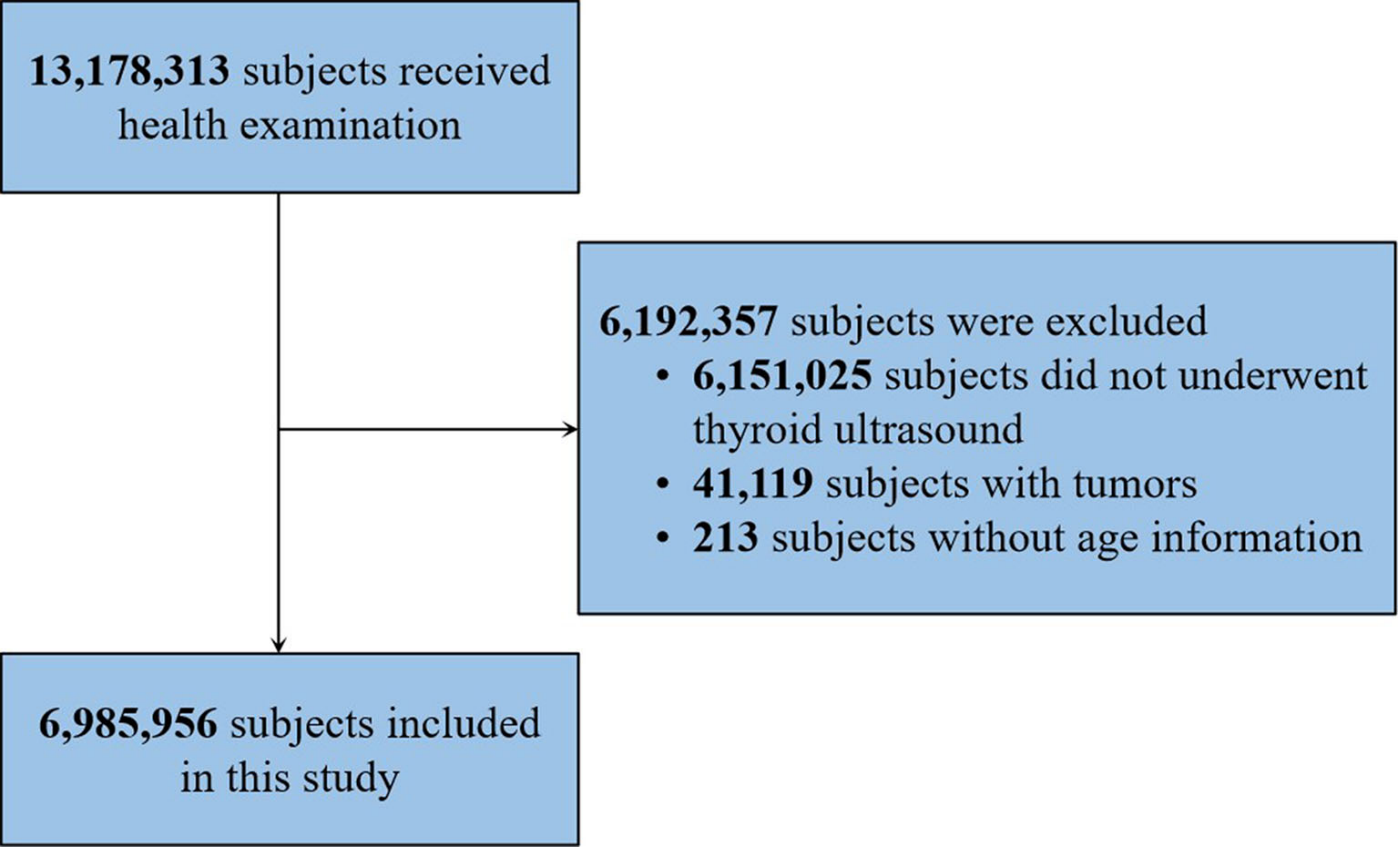
Flowchart of subject selection.

Records of all subjects who received health examinations at Meinian Onehealth Healthcare (MOH) in 2017 were reviewed. MOH (Group) Co., Ltd was founded in 2004 and is a medical group providing preventive health screening services and doctor referrals in China. MOH has more than 600 professional medical centers in mainland China. Between January 1, 2017 and December 31, 2017, 13,178,313 participants received health examinations at MOH with accessible data. Approximately 46.7% (n=6,151,025 individuals) were excluded because they did not undergo thyroid ultrasound examination. Additionally, 41,119 patients with a prior history of malignancy were excluded to avoid influence from malignant disease. Subjects without age information (n=213) were excluded because we suspected that the reliability of the data from these cases was low. Finally, a total of 6,985,956 individuals who received thyroid ultrasound examinations at least once were eligible for this analysis **(** **Figure 1** **)**. Basic information including age, gender, and location were routinely collected for each participant based on a health examination reservation. The province of the individual was defined as the place where he or she received the health examination. This study was reviewed and approved by the Institutional Ethics Committees of the First Affiliated Hospital of Chongqing Medical University, in which written informed consent was waived. No participants received financial compensation.

### Clinical Examination and Laboratory Testing

MOH used uniform criteria for clinical examinations and laboratory testing in all medical centers and guaranteed the uniformity of all health examination results. The thyroid of all included individuals was detected using a thyroid ultrasonography. All ultrasonographies were certified by China Food and Drug Administration (CFDA). Thyroid nodules were defined as discrete lesions in the thyroid gland that were radiologically distinct from the surrounding thyroid parenchyma, including solid, spongiform, cystic, and mixed nodules (1). Physical examination including height (cm), weight (kg), systolic blood pressure (SBP, [mm/Hg]), and diastolic blood pressure (DBP, [mm/Hg]) were measured twice, and averages were recorded. Body mass index (BMI) was calculated as weight (kg) divided by height (m) squared (kg/m^2^). Individuals were grouped into BMI <18.5 kg/m^2^ (underweight), 18.5–23.9 kg/m^2^ (normal), 24.0–27.9 kg/m^2^ (overweight), and ≥28.0 kg/m^2^ (obese) according to the guidelines for prevention and control of overweight and obesity in Chinese adults (13).

Blood samples were obtained by venipuncture after an 8-h fast in the morning. The following factors were measured and available for analysis: triiodothyronine (T3), free triiodothyronine (FT3), thyroxine (T4), free thyroxine (FT4), thyroid-stimulating hormone (TSH), fasting blood glucose (FBG), hemoglobin A1c (HbA1c), uric acid (UA), triglycerides (TG), total cholesterol (TC), high-density lipoprotein (HDL), and low-density lipoprotein (LDL). Participants were classified into high, normal, and low groups based on the normal ranges of these indicators.

### Statistical Analysis

Demographic and baseline characteristics of all participants were analyzed using the chi-square test for categorical variables. The Mann-Whitney U test was used for continuous variables. Unknown data were not included in the analysis. The age- and/or sex-standardized prevalence of thyroid nodules was calculated using the data of the population distribution in China in 2010 by the direct method. Multivariable logistic regression analysis was used to explore potential risk factors associated with thyroid nodules. The model for multivariable logistic regression analysis included the following covariates: age, gender, BMI, SBP, DBP, UA, FBG, TG, HDL, and LDL. Height and weight were removed from the final model to avoid violating the principle of excluding linearly codependent variables because BMI was determined by them. To avoid missing data-related bias, T3, FT3, T4, FT4, TSH, and HbA1c were excluded because of the large amount of missing data. Odds ratios (ORs) and corresponding 95% confidence intervals (CIs) were estimated based on the multivariable model. All tests were 2-sided, and a *P*-value < 0.05 was considered statistically significant. Statistical analyses were performed using SAS software, version 9.2 (SAS Institute Inc, Cary, NC).

## Results

### Study Cohort Characteristics

A total of 6,985,956 individuals, who received thyroid ultrasonography while having a health examination at MOH in 2017, were included in this study **(** **Table 1** **)**. The mean age of the participants was 42.1 years. Among the cases, 3,681,382 (52.7%) were men and 3,304,574 (47.3%) were women. All participants came from 30 provinces and regions of China including Anhui (n=180,624, 2.6%), Beijing (n=191,834, 2.7%), Chongqing (n=64,802, 0.9%), Fujian (n=121,082, 1.7%), Gansu (n=80,582, 1.2%), Guangdong (n=698,583, 10.0%), Guangxi (n=91,999, 1.3%), Guizhou (n=83,877, 1.2%), Hainan (n=40,282, 0.6%), Hebei (n=228,498, 3.3%), Henan (n=542,813, 7.8%), Heilongjiang (n=79,637, 2.6%), Hubei (n=344,377, 2.6%), Hunan (n=140,343, 2.6%), Jilin (n=175,436, 2.6%), Jiangsu (n=360,573, 2.6%), Jiangxi (n=84,497, 1.2%), Liaoning (n=531,872, 7.6%), Inner Mongolia (n=130,261, 1.9%), Ningxia (n=21,837, 0.3%), Qinghai (n=10,461, 0.1%), Shandong (n=627,013, 9.0%), Shanxi (n=143,800, 2.1%), Shaanxi (n=172,691, 2.5%), Shanghai (n=492,928, 7.0%), Sichuan (n=553,015, 7.9%), Tianjin (n=173,734, 2.5%), Xinjiang (n=107,938, 1.5%), Yunnan (n=225,327, 3.2%), and Zhejiang (n=285,240, 4.1%). Thyroid nodules were identified in 2,578,156 participants. Participants with thyroid nodules were significantly older than those without nodules (*P* < 0.001). Thyroid nodules were more frequent in female participants than male (*P* < 0.001). Compared with participants without nodules, heights and weights were significantly lower in the cohort with nodules, while BMI, SBP, and DBP were higher in the group with nodules **(** **Table 1** **)**. There were significant differences in the distributions of thyroid function tests (FT3, FT4, and TSH), glycemic parameters (FBG and HbA1c), UA, and serum lipids (TG, TC, HDL, and LDL) between individuals with and without thyroid nodules **(** **Table 1** **)**.

**Table 1:** Distribution of sociodemographic characteristics and blood factors among subjects with and without thyroid nodules.

Variables	Groups	No nodules		Nodules		*P*-value
		N=4407938	(%)	N=2578231	(%)	
Age		39.14 ± 11.83		47.06 ± 13.65		<0.001
Gender	Male	2579739	58.53%	1101643	42.73%	<0.001
	Female	1828061	41.47%	1476513	57.27%	
Height (cm)		165.95 ± 8.53		163.80 ± 8.57		<0.001
Weight (kg)		66.08 ± 13.17		65.87 ± 12.50		<0.001
BMI (kg/m^2^)		23.87 ± 3.64		24.44 ± 3.53		<0.001
SBP (mm/Hg)		121.46 ± 17.29		126.23 ± 19.78		<0.001
DBP (mm/Hg)		74.80 ± 11.98		76.58 ± 12.41		<0.001
T3	Normal	681457	98.79%	525801	98.76%	<0.001
	Low	4437	0.64%	3696	0.69%	
	High	3920	0.57%	2887	0.54%	
	NA	3717986		2045772		
FT3	Normal	412097	98.18%	391844	98.37%	<0.001
	Low	2079	0.50%	1915	0.48%	
	High	5579	1.33%	4566	1.15%	
	NA	3988045		2179831		
T4	Normal	682284	98.75%	526902	98.77%	0.64
	Low	5144	0.74%	3894	0.73%	
	High	3467	0.50%	2681	0.50%	
	NA	3716905		2044679		
FT4	Normal	412111	97.21%	392587	97.43%	<0.001
	Low	4810	1.13%	4594	1.14%	
	High	7029	1.66%	5760	1.43%	
	NA	3983988		2175290		
TSH	Normal	802817	92.89%	622846	92.01%	<0.001
	Low	11632	1.35%	9624	1.42%	
	High	49815	5.76%	44480	6.57%	
	NA	3543536		1901206		
UA	Normal	3396466	80.60%	2061211	83.72%	<0.001
	Low	20094	0.48%	13873	0.56%	
	High	797173	18.92%	387035	15.72%	
	NA	194067		116037		
FBG	Normal	3961576	92.79%	2207015	88.10%	<0.001
	Low	14580	0.34%	6150	0.25%	
	High	293336	6.87%	292080	11.66%	
	NA	138308		72911		
HbA1c	Normal	414649	90.46%	270613	84.84%	<0.001
	Low	3457	0.75%	1555	0.49%	
	High	40262	8.78%	46791	14.67%	
	NA	3949432		2259197		
TG	Normal	3234098	74.78%	1881437	74.08%	<0.001
	Low	185335	4.29%	94445	3.72%	
	High	905228	20.93%	563856	22.20%	
	NA	83139		38418		
TC	Normal	3494433	80.78%	1947819	76.68%	<0.001
	Low	44880	1.04%	19618	0.77%	
	High	786602	18.18%	572919	22.55%	
	NA	81885		37800		
HDL	Normal	2885236	82.16%	1760487	82.33%	<0.001
	Low	168596	4.80%	94576	4.42%	
	High	457958	13.04%	283396	13.25%	
	NA	896010		439697		
LDL	Normal	2919443	83.31%	1736002	81.32%	<0.001
	Low	107206	3.06%	54228	2.54%	
	High	477757	13.63%	344560	16.14%	
	NA	903394		443366		

BMI, body mass index; SBP, systolic blood pressure; DBP, diastolic blood pressure; T3, triiodothyronine; FT3, free triiodothyronine; T4, thyroxine; FT4, free thyroxine; TSH, thyroid-stimulating hormone; FBG, fasting blood glucose; HbA1c, hemoglobin A1c; UA, uric acid; TG, triglycerides; TC, total cholesterol; HDL, high-density lipoprotein; LDL, low-density lipoprotein; NA, Not available.

### Prevalence of Thyroid Nodules

**Figure 2:**
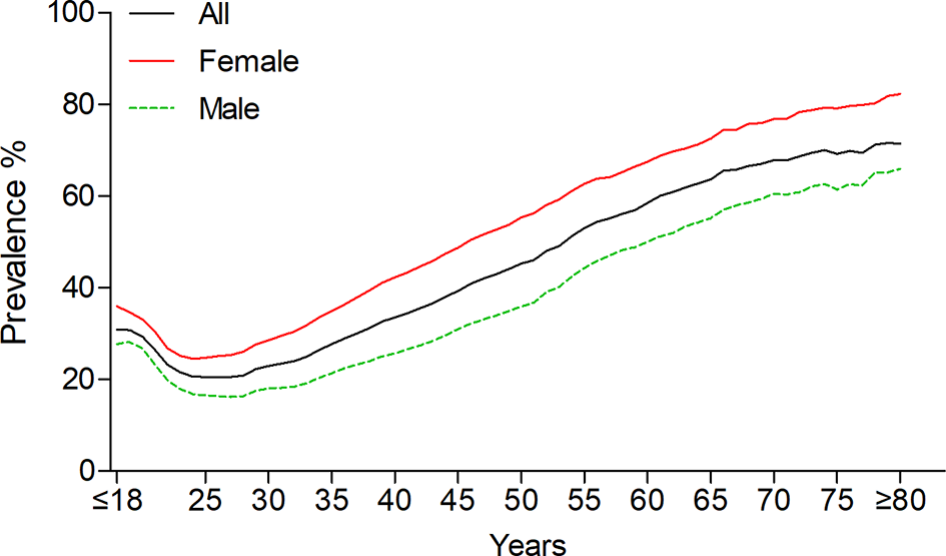
Age-specific prevalence of thyroid nodules.

**Figure 3:**
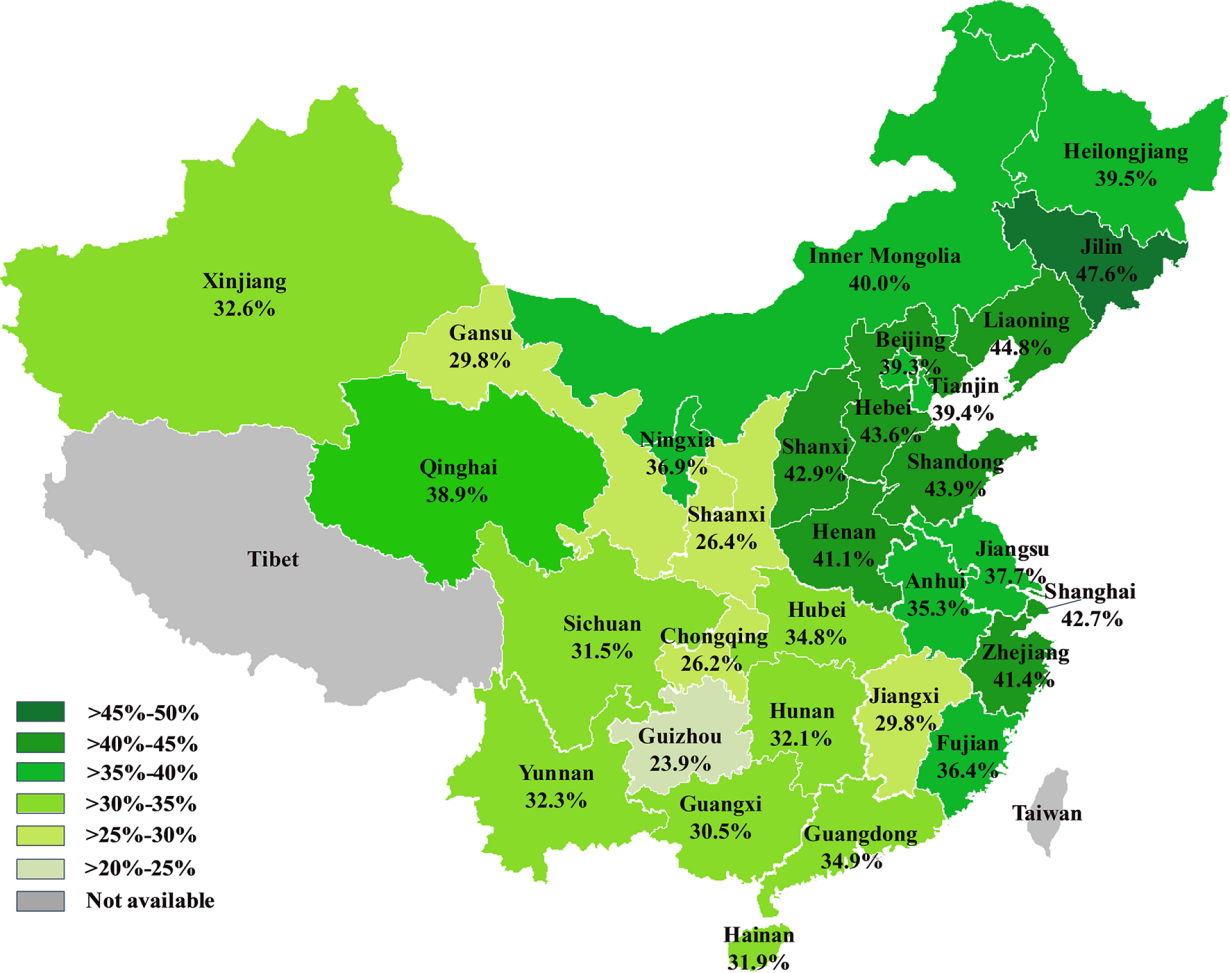
The prevalence of thyroid nodules in 30 provinces and regions of China.

**Figure 4:**
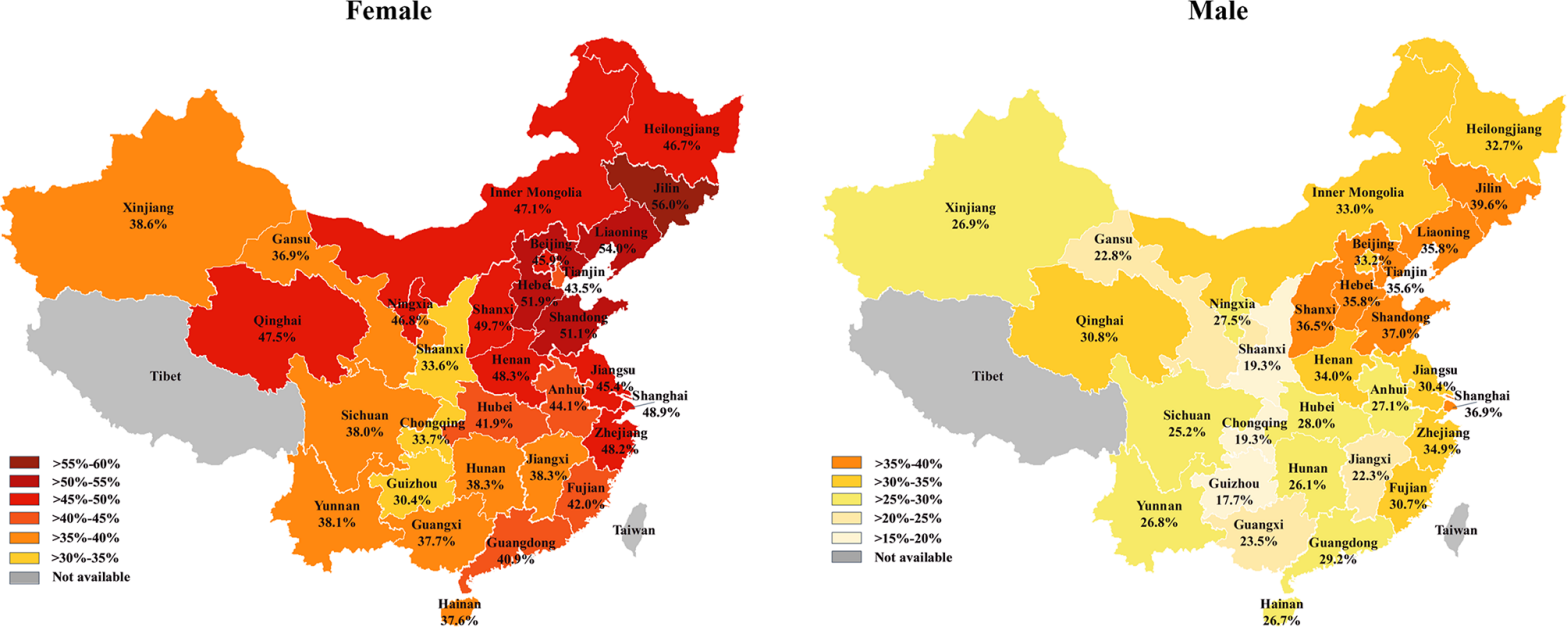
The prevalence of thyroid nodules in females (left) and males (right) in China.

The overall prevalence of thyroid nodules was 36.9% (95% CI, 35.7%–38.1%). After standardizing by age and sex based on the population of China in 2010, the adjusted prevalence was 38.0% (95% CI, 37.0%–39.1%). The crude nodule prevalence among female subjects was 44.7% (95% CI, 43.4%–45.9%; age-standardized: 45.2% [95% CI, 44.1%–46.4%]), which was significantly higher than males (29.9% [95% CI, 28.8%–31.0%]; age-standardized: 31.2% [95% CI, 30.1%–32.2%]); *P* < 0.001). When analyzed by age, the prevalence of thyroid nodules was decreased in participants aged from ≤18 (30.8%) to 26 (20.5%), but increased over 26 years old in the overall cohort, up to 71.4% in subjects ≥80 years old **(** **Figure 2** **)**. In women, the prevalence was decreased from ≤18 (35.9%) to 24 (24.5%), and increased after 24 up to maximum 82.2% in female participants ≥80 years. In men, the prevalence was lowest at 27 years old (16.2%) and gradually increased to 65.9% in participants ≥80 years. Among the 30 provinces and regions, the standardized prevalence of thyroid nodules ranged from 23.9% (Guizhou) to 47.6% (Jilin) in the overall cohort **(** **Figure 3** **)**. Similar trends of the standardized prevalence were observed after stratifying individuals by gender **(** **Figure 4** **)**. In most provinces and regions, women had 10% higher prevalence of thyroid nodules than men, except for Tianjin, where women had an 8.0% higher prevalence rate. Ningxia had the largest difference of nodule prevalence between females and males (19.3%), followed by Liaoning (18.2%) and Anhui (17.0%). The northeast of China had the highest nodule prevalence (46.8% [95% CI, 44.1%–49.2%]) followed by the north (41.2% [95% CI, 39.5%–42.9%]), east (38.6% [95% CI, 36.6%–40.5%]), central (37.4% [95% CI, 34.8%–40.0%]), south (30.5% [95% CI, 28.3%–32.7%]), southwest (29.3% [95% CI, 26.9%–31.6%]), and northwest (28.9% [95% CI, 26.9%–31.6%]). Coastal provinces had a significantly higher prevalence than inland provinces (38.9% [95% CI, 37.5%–41.0%] vs. 34.5% [95% CI, 33.1%–36.0%]; *P* < 0.001).

### Baseline Variables Associated With Thyroid Nodules

To determine factors related to thyroid nodules, we fit a multivariable logistic regression model, including age, gender, BMI, SBP, DBP, UA, FBG, TG, HDL, and LDL among the entire cohort **(** **Table 2** **)**. Compared with individuals aged ≤18–25, the odds of thyroid nodules were significantly increased with age >25 years. However, there was no obvious difference between the ≤18–25 and 26–40 groups in men (OR, 1.01; 95% CI, 1.00–1.02; *P* = 0.07). Women had more than 2-fold higher odds (OR, 2.22; 95% CI, 2.19–2.21; *P* < 0.001) compared with men. Individuals with BMI, SBP, DBP, and FBG values below the normal range were associated with significantly less odds of thyroid nodules, whereas those with higher BMI, SBP, DBP, and FBG were significantly associated with a greater risk of thyroid nodules. High UA levels were significantly associated with lower risk of thyroid nodules (OR, 0.98; 95% CI, 0.98–0.99; *P* < 0.001). Compared with participants who had normal levels of TG and HDL, TG presents a U-shape association with risk of thyroid nodules, whereas abnormal levels of HDL were significantly associated with decreased risk of thyroid nodules **(** **Table 2** **)**. Low vs. normal LDL levels were significantly associated with decreased odds of thyroid nodules in the overall cohort (OR, 0.86; 95% CI, 0.85–0.87; *P* < 0.001), as well as women (OR, 0.89; 95% CI, 0.88–0.90; *P* < 0.001) and men (OR, 0.82; 95% CI, 0.81-0.84; *P* < 0.001). High vs. normal LDL levels were associated with an increased risk of thyroid nodules in women (OR, 1.04; 95% CI, 1.03–1.04; *P* < 0.001); however, a decreased risk was observed in men with high LDL levels (OR, 0.98; 95% CI, 0.97–0.98; *P* < 0.001).

**Table 2:** Multivariable logistic regression analysis.

Variables		All		Female		Male	
		OR (95% CI)	P–value	OR (95% CI)	P–value	OR (95% CI)	P–value
Age	≤18–25	reference		reference		reference	
	26–40	1.16 (1.15–1.17)	<0.001	1.29 (1.27–1.30)	<0.001	1.01 (1.00–1.02)	0.07
	41–55	2.39 (2.38–2.41)	<0.001	2.74 (2.71–2.77)	<0.001	2.02 (1.99–2.04)	<0.001
	56–70	4.61 (4.59–4.67)	<0.001	5.02 (4.96–5.08)	<0.001	4.08 (4.03–4.12)	<0.001
	≥71	7.59 (7.49–7.69)	<0.001	8.48 (8.30–8.66)	<0.001	6.58 (6.47–4.12)	<0.001
Gender	Male	reference		NA		NA	
	Female	2.20 (2.19–2.21)	<0.001	NA		NA	
BMI	<18.5	0.77 (0.77–0.78)	<0.001	0.77 (0.77–0.78)	<0.001	0.80 (0.79–0.82)	<0.001
	18.5–23.9	reference		reference		reference	
	24.0–27.9	1.20 (1.19–1.20)	<0.001	1.20 (1.19–1.20)	<0.001	1.20 (1.19–1.21)	<0.001
	≥28.0	1.32 (1.32–1.33)	<0.001	1.30 (1.29–1.31)	<0.001	1.34 (1.33–1.35)	<0.001
	Unknown	1.03 (1.02–1.04)	<0.001	0.99 (0.88–1.01)	0.38	1.07 (1.06–1.08)	<0.001
SBP	<90	0.90 (0.88–0.92)	<0.001	0.90 (0.88–0.92)	<0.001	0.92 (0.86–0.98)	0.01
	90–139	reference		reference		reference	
	≥140	1.13 (1.12–1.14)	<0.001	1.17 (1.16–1.18)	<0.001	1.10 (1.09–1.11)	<0.001
	Unknown	1.16 (0.96–1.40)	0.13	1.14 (0.92–1.42)	0.23	1.25 (0.85–1.84)	0.25
DBP	<60	0.91 (0.91–0.92)	<0.001	0.92 (0.91–0.92)	<0.001	0.92 (0.90–0.93)	<0.001
	60–89	reference		reference		reference	
	≥90	1.05 (1.05–1.06)	<0.001	1.07 (1.06–1.09)	<0.001	1.06 (1.05–1.07)	<0.001
	Unknown	0.93 (0.77–1.13)	0.48	0.96 (0.77–1.19)	0.71	0.86 (0.59–1.27)	0.45
UA	Normal	reference		reference		reference	
	Low	0.99 (0.97–1.02)	0.66	0.99 (0.99–1.02)	0.40	1.00 (0.97–1.04)	0.83
	High	0.98 (0.98–0.99)	<0.001	0.98 (0.97–0.99)	<0.001	0.98 (0.98–0.99)	<0.001
	Unknown	1.06 (1.05–1.18)	<0.001	1.06 (1.05–1.07)	<0.001	1.04 (1.03–1.06)	<0.001
FBG	Normal	reference		reference		reference	
	Low	0.94 (0.918–0.97)	<0.001	0.93 (0.89–0.97)	<0.001	0.95 (0.90–0.99)	0.02
	High	1.17 (1.16–1.18)	<0.001	1.17 (1.16–1.18)	<0.001	1.18 (1.17–1.19)	<0.001
	Unknown	0.99 (0.98–1.00)	.42	1.00 (0.99–1.02)	0.77	0.97 (0.96–0.99)	<0.001
TG	Normal	reference		reference		reference	
	Low	1.05 (1.04–1.06)	<0.001	1.03 (1.02–1.04)	<0.001	1.07 (1.06–1.09)	<0.001
	High	1.02 (1.01–1.02)	<0.001	1.04 (1.03–1.05)	<0.001	1.01 (1.01–1.02)	<0.001
	Unknown	0.90 (0.89–0.91)	<0.001	0.91 (0.89–0.93)	<0.001	0.89 (0.87–0.91)	<0.001
HDL	Normal	reference		reference		reference	
	Low	0.98 (0.97–0.98)	<0.001	0.94 (0.92–0.95)	<0.001	0.99 (0.98–1.00)	0.05
	High	0.90 (0.90–0.91)	<0.001	0.94 (0.93–0.95)	<0.001	0.85 (0.84–0.85)	<0.001
	Unknown	1.02 (0.98–1.06)	0.31	1.02 (0.96–1.07)	0.57	1.02 (0.96–1.07)	0.55
LDL	Normal	reference		reference		reference	
	Low	0.86 (0.85–0.87)	<0.001	0.89 (0.88–0.90)	<0.001	0.82 (0.81–0.84)	<0.001
	High	1.00 (1.00–1.01)	0.23	1.04 (1.03–1.04)	<0.001	0.98 (0.97–0.98)	<0.001
	Unknown	0.91 (0.88–0.95)	<0.001	0.93 (0.88–0.98)	0.004	0.91 (0.86–0.96)	<0.001

BMI, body mass index; SBP, systolic blood pressure; DBP, diastolic blood pressure; FBG, fasting blood glucose; UA, uric acid; TG, triglycerides; HDL, high-density lipoprotein; LDL, low-density lipoprotein; NA, Not available.

## Discussion

In this study, we systematically analyzed the prevalence of thyroid nodules among a huge cohort from 30 provinces and regions of China and revealed that the overall prevalence of thyroid nodules was 38.0%. This is close to the prevalence in Korea (34.2%), a neighboring country of China (14). However, we found that the prevalence of thyroid nodules varied from different provinces in China in a regional-specific manner. The northeast (Liaoning, Jilin, and Heilongjiang) and north (Beijing, Tianjin, Hebei, and Inner Mongolia) of China had a higher prevalence (over 40%), whereas the southwest (Sichuan, Guizhou, Chongqing, and Yunnan) and northwest (Shaanxi, Ningxia, Gansu, Qinghai, and Xinjiang) of China had a lower prevalence (approximately 30%). This prevalence pattern was different from a previous meta-analysis that pooled data between 2006 and 2013 in China (12). Additionally, the nodule prevalence of coastal provinces was higher than inland provinces. This regional-specific prevalence might result from multiple causes such as inheritance, environmental factors, and lifestyle.

Liaoning province had the second highest prevalence of thyroid nodules (44.8%) in China, and our data suggested it was much higher than data reported in 2008 (approximately 10%) and 2009 (38.5%) (15, 16). The prevalence of thyroid nodules in Shanxi province was also higher in our study than in a previous study (17). Jiang et al. reported that the prevalence of thyroid nodules in Beijing was 40.1% based on 6,324 participants in 2016, which was close to our result (18). In Zhejiang province, the reported prevalence of thyroid nodules has varied from 22.5% to 38.4% (12, 19), which was lower than the prevalence of 41.4% in this study (n=285,240 individuals from Zhejiang). However, these discrepancies cannot be interpreted as changes in the inheritance and environmental factors, but be dramatic changes of lifestyle over past decade. In addition, the different sampling methods might also have impacted these results. Nevertheless, due to the reliability of our study population and the considerable number of participants, we believe that the present study is more reliable in reflecting the actual prevalence of thyroid nodules in China.

Gender and age are two critical factors associated with the prevalence of thyroid nodules (20). Consistent with previous studies, the frequency of thyroid nodules was significantly higher in female than that in male participants (14, 18, 21). However, it was unexpected that the age-specific prevalence of thyroid nodules was decreased with age from ≤18 to 26 years and increased in those older than 26 years, a phenomenon that was distinct from other studies, which concluded that the frequency of thyroid nodules increases with age (14, 18, 20–22). These reports might have missed this phenomenon because of limitations of sample size and grouping individuals by decade, whereas our result was supported by an investigation that included a relatively large cohort (17). In the study by Liu et al., they found that the frequency of nodules showed a U-shaped curve with age increase and the group aged 20–30 years had the lowest prevalence (17). Together, these results indicated that the events of thyroid nodule disappearance exceed new nodules development during the age ≤18–26 years. In contrast, a recent study that included 992 thyroid nodule patients with mean age 52.4 years and 5-year follow-up, showed that new thyroid nodules occurred in 93 (9.3%) patients, whereas only one nodule disappeared during follow-up (5). However, further studies focused on young individuals are warranted to reveal the natural history of thyroid nodules and to clarify the underlying mechanisms with respect to thyroid nodules that spontaneously shrink or disappear.

The multivariable logistic regression analysis indicated that age and gender were two independent risk factors associated with the development of thyroid nodules, which was consistent with other studies (21). Women had a 2.2-fold increased risk of developing thyroid nodules than men. Moreover, previous studies have reported that pregnancy was associated with new thyroid nodule formation and increased nodule size (23, 24). These results suggested that the development of thyroid nodules may be influenced by sex-related hormones such as estrogen and progesterone. Higher BMI was associated with an increased risk of thyroid nodules, whereas participants with a BMI lower than normal had a decreased risk of nodules. High FBG levels were an independent risk factor for thyroid nodules. It was consistent with the finding that thyroid nodule development is associated with obesity, insulin resistance, prediabetes, and diabetes (25–28). In addition, central obesity, defined by a gender-specific waist circumference cut-off, has been demonstrated to be another independent risk factor for thyroid nodules (29). In this study, we found that blood pressure, UA, and serum lipids (TG, HDL, and LDL) were significantly associated with nodule disease. Although other studies have also shown similar relationships of thyroid nodules with hypertension, hyperuricemia, and serum lipids, the underlying mechanisms through which hypertension and serum lipids promote the development of thyroid nodules remain unclear (27, 30, 31).

Iodine intake has been suspected to be a major dietary factor affecting the development of thyroid nodules, but the evidence remains inconclusive. In 1995, to prevent iodine deficiency disorders, the Chinese government launched a Universal Salt Iodization program, which directly resulted in a dramatic decrease of goiters. However, the number of cases with thyroid nodules, thyroid cancer, and hyperthyroidism increased over the same period. Therefore, some experts hold the point of view that iodine intake might be excessive in the Chinese population, especially in coastal provinces. Nevertheless, the reality is that individuals with deficient, adequate, and excessive iodine intake have prevalence of thyroid nodules 23.7%, 53.8% and 18.0%, respectively, as reported by a previous study (32). Participants with high or excessive iodine intake did not show an increased risk of thyroid nodules, whereas deficient iodine intake was significantly associated with an increased risk of nodules (32). Similarly, participants consuming iodized salt had a lower prevalence of thyroid nodules than those consuming non-iodized salt (21). However, they found that the prevalence of thyroid nodules increased when the urinary iodine concentrations were >500 μg/L. Regrettably, data regarding iodized salt intake and urinary iodine concentrations were not available for this study. Although we found a significant difference in the nodule prevalence between coastal and inland provinces, this cannot be simply explained by eating seafood, which is rich in iodine. Therefore, further investigations into the relationship between iodine and thyroid nodules are needed in the future.

This study had several limitations. First, the participants who received health examinations at MOH cannot represent the overall Chinese population due available data from real world health screening practice. In addition, 46.7% participants without thyroid ultrasound examination were excluded. These may result in sample selection bias. Second, the size, number and ultrasound imaging characteristics of thyroid nodules were not recorded in all medical records when we extracted thyroid nodules using a computerized keyword extraction method, we could not analyze nodule size, nodule number, and ultrasound imaging characteristics, such as microcalcification and sonogram echoes. Third, there were no biopsy data for sonographically suspicious nodules or follow-up for all individual included in this study. Therefore, the proportion of thyroid cancer among these nodules and the natural history of the thyroid nodules were unclear. Despite these limitations, to our knowledge, this study represents the first investigation of the prevalence of thyroid nodules nationwide in China using a huge sample size. We believe that these results appropriately reflect the disease status of thyroid nodules in China.

In summary, the results of this study suggested that the prevalence of thyroid nodules was high in the Chinese population and showed a regional-specific pattern. Thyroid nodules were also more prevalent among women than men. The prevalence of thyroid nodules was decreased from ≤18 to 26 years and increased with age >26 years in the overall cohort. Factors including age, gender, BMI, SBP, DBP, UA, FBG, TG, HDL, and LDL were independently associated with development of thyroid nodules. These findings enhance our understanding of thyroid nodules in China.

## Data Availability Statement

The raw data supporting the conclusions of this article will be made available by the authors, without undue reservation.

## Ethics Statement

The studies involving human participants were reviewed and approved by Institutional Ethics Committees of the First Affiliated Hospital of Chongqing Medical University. Written informed consent from the participants’ legal guardian/next of kin was not required to participate in this study in accordance with the national legislation and the institutional requirements.

## Author Contributions

GR and YL designed the study. GR and YN supervised the study. YN, CJ, and MT had full access to all of the data in the study. CJ and MT conducted the statistical analysis. YL, JL, and MW drafted the manuscript. JH and YN revised the manuscript. All authors contributed to the article and approved the submitted version.

## Conflict of Interest

The authors declare that the research was conducted in the absence of any commercial or financial relationships that could be construed as a potential conflict of interest.

## References

[1] Haugen BR Alexander EK Bible KC Doherty GM Mandel SJ Nikiforov YE. 2015 American Thyroid Association Management Guidelines for Adult Patients With Thyroid Nodules and Differentiated Thyroid Cancer: The American Thyroid Association Guidelines Task Force on Thyroid Nodules and Differentiated Thyroid Cancer. Thyroid (2016) 26(1):1–133. 10.1089/thy.2015.0020 26462967 PMC4739132

[2] Valderrabano P McGettigan MJ Lam CA Khazai L Thompson ZJ Chung CH. Thyroid Nodules With Indeterminate Cytology: Utility of the American Thyroid Association Sonographic Patterns for Cancer Risk Stratification. Thyroid (2018) 28(8):1004–12. 10.1089/thy.2018.0085 PMC6916126 29848195

[3] Tan GH Gharib H. Thyroid Incidentalomas: Management Approaches to Nonpalpable Nodules Discovered Incidentally on Thyroid Imaging. Ann Intern Med (1997) 126(3):226–31. 10.7326/0003-4819-126-3-199702010-00009 9027275

[4] Durante C Grani G Lamartina L Filetti S Mandel SJ Cooper DS. The Diagnosis and Management of Thyroid Nodules: A Review. Jama (2018) 319(9):914–24. 10.1001/jama.2018.0898 29509871

[5] Durante C Costante G Lucisano G Bruno R Meringolo D Paciaroni A. The Natural History of Benign Thyroid Nodules. Jama (2015) 313(9):926–35. 10.1001/jama.2015.0956 25734734

[6] Dralle H Machens A Basa J Fatourechi V Franceschi S Hay ID. Follicular Cell-Derived Thyroid Cancer. Nat Rev Dis Primers (2015) 1:15077. 10.1038/nrdp.2015.77 27188261

[7] Burman KD Wartofsky L. Clinical Practice. Thyroid Nodules. N Engl J Med (2015) 373(24):2347–56. 10.1056/NEJMcp1415786 26650154

[8] Wong R Farrell SG Grossmann M. Thyroid Nodules: Diagnosis and Management. Med J Aust (2018) 209(2):92–8. 10.5694/mja17.01204 29996756

[9] Bibbins-Domingo K Grossman DC Curry SJ Barry MJ Davidson KW Doubeni CA. Screening for Thyroid Cancer: US Preventive Services Task Force Recommendation Statement. Jama (2017) 317(18):1882–7. 10.1001/jama.2017.4011 28492905

[10] Sosa JA Duh QY Doherty G. Striving for Clarity About the Best Approach to Thyroid Cancer Screening and Treatment: Is the Pendulum Swinging Too Far? JAMA Surg (2017) 152(8):721–2. 10.1001/jamasurg.2017.1338 28492919

[11] Danese D Sciacchitano S Farsetti A Andreoli M Pontecorvi A. Diagnostic Accuracy of Conventional Versus Sonography-Guided Fine-Needle Aspiration Biopsy of Thyroid Nodules. Thyroid (1998) 8(1):15–21. 10.1089/thy.1998.8.15 9492148

[12] Zhao W Han C Shi X Xiong C Sun J Shan Z. Prevalence of Goiter and Thyroid Nodules Before and After Implementation of the Universal Salt Iodization Program in Mainland China From 1985 to 2014: A Systematic Review and Meta-Analysis. PloS One (2014) 9(10):e109549. 10.1371/journal.pone.0109549 25313993 PMC4196906

[13] Chen C Lu FC. The Guidelines for Prevention and Control of Overweight and Obesity in Chinese Adults. BioMed Environ Sci (2004) 17 Suppl:1–36. 10.1111/j.1365-2028.2008.00991.x 15807475

[14] Moon JH Hyun MK Lee JY Shim JI Kim TH Choi HS. Prevalence of Thyroid Nodules and Their Associated Clinical Parameters: A Large-Scale, Multicenter-Based Health Checkup Study. Korean J Intern Med (2018) 33(4):753–62. 10.3904/kjim.2015.273 PMC6030422 28859466

[15] Pan XF Sun XY Jia XD Xu F Zhao T Jiang T. Thyroid Nodular Diseases in the Population Indergoing Medical Examination and the Analysis of its Relative Factors in Dalian City,Liaoning Province. Chin J Endemiol (2009) 28(5):568–71. 10.3760/cma.j.issn.1000-4955.2009.05.030

[16] Yu X Fan C Shan Z Teng X Guan H Li Y. A Five-Year Follow-Up Study of Goiter and Thyroid Nodules in Three Regions With Different Iodine Intakes in China. J Endocrinol Invest (2008) 31(3):243–50. 10.1007/bf03345597 18401207

[17] Liu Y Lin Z Sheng C Zhu Y Huang Y Zhong N. The Prevalence of Thyroid Nodules in Northwest China and its Correlation With Metabolic Parameters and Uric Acid. Oncotarget (2017) 8(25):41555–62. 10.18632/oncotarget.14720 PMC5522227 28107199

[18] Jiang H Tian Y Yan W Kong Y Wang H Wang A. The Prevalence of Thyroid Nodules and an Analysis of Related Lifestyle Factors in Beijing Communities. Int J Environ Res Public Health (2016) 13(4):442. 10.3390/ijerph13040442 27110805 PMC4847104

[19] Xu W Chen Z Li N Liu H Huo L Huang Y. Relationship of Anthropometric Measurements to Thyroid Nodules in a Chinese Population. BMJ Open (2015) 5(12):e008452. 10.1136/bmjopen-2015-008452 PMC4691709 26692553

[20] Mazzaferri EL. Management of a Solitary Thyroid Nodule. N Engl J Med (1993) 328(8):553–9. 10.1056/nejm199302253280807 8426623

[21] Fan L Tan L Chen Y Du C Zhu M Wang K. Investigation on the Factors That Influence the Prevalence of Thyroid Nodules in Adults in Tianjin, China. J Trace Elem Med Biol (2018) 50:537–42. 10.1016/j.jtemb.2018.03.004 29544745

[22] Kwong N Medici M Angell TE Liu X Marqusee E Cibas ES. The Influence of Patient Age on Thyroid Nodule Formation, Multinodularity, and Thyroid Cancer Risk. J Clin Endocrinol Metab (2015) 100(12):4434–40. 10.1210/jc.2015-3100 PMC4667162 26465395

[23] Kung AW Chau MT Lao TT Tam SC Low LC. The Effect of Pregnancy on Thyroid Nodule Formation. J Clin Endocrinol Metab (2002) 87(3):1010–4. 10.1210/jcem.87.3.8285 11889153

[24] Alexander EK Pearce EN Brent GA Brown RS Chen H Dosiou C. Guidelines of the American Thyroid Association for the Diagnosis and Management of Thyroid Disease During Pregnancy and the Postpartum. Thyroid (2017) 27(3):315–89. 10.1089/thy.2016.0457 28056690

[25] Anil C Akkurt A Ayturk S Kut A Gursoy A. Impaired Glucose Metabolism is a Risk Factor for Increased Thyroid Volume and Nodule Prevalence in a Mild-to-Moderate Iodine Deficient Area. Metabolism (2013) 62(7):970–5. 10.1016/j.metabol.2013.01.009 23395200

[26] Sousa PA Vaisman M Carneiro JR Guimaraes L Freitas H Pinheiro MF. Prevalence of Goiter and Thyroid Nodular Disease in Patients With Class III Obesity. Arq Bras Endocrinol Metabol (2013) 57(2):120–5. 10.1590/S0004-27302013000200004 23525289

[27] Feng S Zhang Z Xu S Mao X Feng Y Zhu Y. The Prevalence of Thyroid Nodules and Their Association With Metabolic Syndrome Risk Factors in a Moderate Iodine Intake Area. Metab Syndr Relat Disord (2017) 15(2):93–7. 10.1089/met.2016.0077 27929732

[28] Buscemi S Massenti FM Vasto S Galvano F Buscemi C Corleo D. Association of Obesity and Diabetes With Thyroid Nodules. Endocrine (2018) 60(2):339–47. 10.1007/s12020-017-1394-2 28836113

[29] Song B Zuo Z Tan J Guo J Teng W Lu Y. Association of Thyroid Nodules With Adiposity: A Community-Based Cross-Sectional Study in China. BMC Endocr Disord (2018) 18(1):3. 10.1186/s12902-018-0232-8 29374470 PMC5787304

[30] Shin J Kim MH Yoon KH Kang MI Cha BY Lim DJ. Relationship Between Metabolic Syndrome and Thyroid Nodules in Healthy Koreans. Korean J Intern Med (2016) 31(1):98–105. 10.3904/kjim.2016.31.1.98 26767863 PMC4712440

[31] Ding X Xu Y Wang Y Li X Lu C Su J. Gender Disparity in the Relationship Between Prevalence of Thyroid Nodules and Metabolic Syndrome Components: The SHDC-CDPC Community-Based Study. Mediators Inflamm (2017) 2017:8481049–. 10.1155/2017/8481049 PMC5457761 28607535

[32] Chen Z Xu W Huang Y Jin X Deng J Zhu S. Associations of Noniodized Salt and Thyroid Nodule Among the Chinese Population: A Large Cross-Sectional Study. Am J Clin Nutr (2013) 98(3):684–92. 10.3945/ajcn.112.054353 23842457

